# Change Mechanisms of Schema-Centered Group Psychotherapy with Personality Disorder Patients

**DOI:** 10.1371/journal.pone.0039687

**Published:** 2012-06-22

**Authors:** Wolfgang Tschacher, Peter Zorn, Fabian Ramseyer

**Affiliations:** Department of Psychotherapy, University Hospital of Psychiatry, Bern, Switzerland; Catholic University of Sacred Heart of Rome, Italy

## Abstract

**Background:**

This study addressed the temporal properties of personality disorders and their treatment by schema-centered group psychotherapy. It investigated the change mechanisms of psychotherapy using a novel method by which psychotherapy can be modeled explicitly in the temporal domain.

**Methodology and Findings:**

69 patients were assigned to a specific schema-centered behavioral group psychotherapy, 26 to social skills training as a control condition. The largest diagnostic subgroups were narcissistic and borderline personality disorder. Both treatments offered 30 group sessions of 100 min duration each, at a frequency of two sessions per week. Therapy process was described by components resulting from principal component analysis of patients' session-reports that were obtained after each session. These patient-assessed components were Clarification, Bond, Rejection, and Emotional Activation. The statistical approach focused on time-lagged associations of components using time-series panel analysis. This method provided a detailed quantitative representation of therapy process. It was found that Clarification played a core role in schema-centered psychotherapy, reducing rejection and regulating the emotion of patients. This was also a change mechanism linked to therapy outcome.

**Conclusions/Significance:**

The introduced process-oriented methodology allowed to highlight the mechanisms by which psychotherapeutic treatment became effective. Additionally, process models depicted the actual patterns that differentiated specific diagnostic subgroups. Time-series analysis explores Granger causality, a non-experimental approximation of causality based on temporal sequences. This methodology, resting upon naturalistic data, can explicate mechanisms of action in psychotherapy research and illustrate the temporal patterns underlying personality disorders.

## Introduction

The larger part of contemporary psychotherapy research has its focus no longer on the efficacy of certain approaches, but on investigating the processes by which psychotherapy becomes effective. A number of scientific methods are feasible in research that pursues such process goals. The basis of process research is exploratory: Many researchers as well as clinicians have used a variety of empirical approaches, qualitative and quantitative, and investigated linguistic material or video recordings of therapy sessions. Consequently, candidates for process factors and change mechanisms of psychotherapy were identified. Exploratory research has yielded numerous process variables that are of general importance for psychotherapy. Dozens of relevant process factors have risen from a half-century of process-outcome literature [1], which consists of studies that are in their majority neither randomized nor experimental. The same is true with respect to the issue of instrumental factors of psychotherapy that exist across the different psychotherapy modalities [2], [3]: Theoretical considerations and reviews of the available process research have yielded a considerable number of (generally accepted) ‘common factors’. Their number likely exceeds 20 [4].

Subsequent to collecting candidate process factors, a next step should be to proceed to more rigorous, hypothesis-driven methods that allow to identify those ingredients of process that are causally responsible for psychotherapy-induced change in a given context. In principle, randomized controlled trials (RCT) have this capacity. RCTs, however, are expensive and difficult to realize in many clinical settings. RCTs are especially bothersome when the intervention to be investigated is group psychotherapy because randomized allocation to group treatment is an even greater logistic challenge than randomized allocation to individual treatment, so that the former is often not viable in centers unless these have high admission numbers. It is therefore reasonable to consider alternative methods to investigate causal mechanisms. One possible solution is to utilize the temporal flow of psychotherapy more explicitly, since psychotherapy process is an instantiation of communicational dynamics [5]. Quasi-causal inferences may be drawn on the basis of Granger causality, employing time-lagged correlations, and this may help identify change mechanisms even in naturalistic process data [6]. It is thus feasible to address change mechanisms beyond RCT methodology, for which purpose time-series analysis appears to be particularly appropriate.

The presence of personality disorder (PD) is defined by current classification systems (e.g. the Diagnostic and Statistical Manual of Mental Disorders, DSM-IV-TR) as an “enduring pattern of inner experience and behavior” that deviates from cultural and societal conventions and expectations, and entails emotion-regulation problems and dysfunctional interpersonal behavior. The DSM distinguishes three clusters of PD (cluster A: odd, eccentric disorders; B: dramatic, emotional or erratic disorders; C: anxious, fearful disorders). A psychodynamic view of the psychopathology encountered in PD was developed by Kernberg's theory of the personality organization in borderline PD [7]. The pathology is seen as residing in problematic relations with inner objects. Since the 1990s, cognitive-behavioral psychotherapy increasingly also developed treatments for PD, starting with dialectical behavior therapy [8]. Schema-focused therapy was a further ‘third-wave’ cognitive psychotherapy, here especially focused on the maladaptive interpersonal and self schemas [9]. The schema concept is obviously reminiscent of the object relations developed by psychodynamic theorists. Schemas become instrumental as mediators of past experiences, and thereby may produce the recurrent patterns found in PD patients [10].

It has been shown that such psychotherapeutic approaches – which may differ with respect to their theoretical underpinnings but converge with respect to the concepts deemed essential – are efficacious in the treatment of PD. Most efficacy studies have addressed borderline PD, where both psychodynamic and dialectical behavior therapy resulted in positive change of psychopathology [11]. A meta-analysis [12] supported findings that psychodynamic as well as cognitive behavior therapy are effective treatments of PD. An integrative cognitive-behavioral group therapy, developed for different categories of PD (SET: Schema-centered emotive-behavior therapy), was found to be effective in a controlled evaluation study [13].

Despite the demonstrated efficacy, the field is unsure about the underlying mechanisms. In the extensive literature on process-outcome associations, clear evidence speaks for the significance of a favorable *therapeutic bond* [1], [14], the single predictor of outcome that has received abundant empirical support. *Clarification/insight* is viewed as a further common factor that entails therapeutic success [4]. Especially insight-oriented psychotherapy approaches, with psychoanalysis and experiential therapy as prototypes, propose that clarification of patients' problems is a major factor of change. Cognitive shift and insight were both conceptualized as ‘in-session impacts’ in several process-outcome studies of recent decades, with a clear predominance of findings pointing towards their positive associations with outcome (p. 357f in [1]). A third general assumption in psychotherapy research is that the emotionality of patients is essential, especially in Cluster-B personality disorder [15], [16]. This common factor of *emotional activation* of patients, emphasized by the humanistic approach in psychotherapy, is also addressed by Grawe's actuation of problems [17]. Lastly, *group cohesion* is an acknowledged change factor in psychotherapeutic group settings [18].

The basic assumption of the present project was that, whenever sufficient data on the temporal patterns in psychotherapy process are available, the detection of change mechanisms in PD treatment may be greatly facilitated. Studies of temporal patterns in PD have extended over differing periods of time, ranging from several years in longitudinal studies [19] to hours or less in ambulatory-assessment approaches of emotion regulation [20]. In the present project, we quantified PD patients' intermediate process patterns, covering a period of up to four months. In the context of psychotherapeutic interventions, this period constitutes a natural time window in which a complete course of treatment can be observed. When the measurement of this period is repeated frequently, at appropriately distanced points in time, time-series statistics [21] can be used to fit models that characterize the process of therapy. Temporal trajectories of, and lagged associations between, repeated measurements can then open up a fine-grained and direct view on change mechanisms of psychotherapeutic treatment.

## Methods

### Objectives

In the present project, change-related components were captured using therapy session reports. We monitored the patterns of temporal sequences of components using a process design. The main goal of the present study was to investigate such patterns in a large dataset that provided a detailed depiction of patients with PD who received schema-oriented group psychotherapy of the SET approach. Monitoring took place after each of the therapy sessions allowing to model session-by-session process. In the first place, we hypothesized that specific process models would underlie psychotherapy in these patients: What was the mechanism of action in SET psychotherapy? Our hypothesis was that SET should specifically draw on the clarification of patients' problems. A further goal was to model any potential differences in process models attributable to the different categories of PD. We finally examined the process models in the context of therapy outcome, performing process-outcome analyses under the hypothesis that aspects of therapy process were significantly associated with treatment outcome.

### Participants

All participants of this study were patients who had received the diagnosis of a personality disorder (PD) according to DSM-IV; SKID-II interviews [22] were conducted to determine the primary diagnosis. SKID-II is a two-step procedure consisting of self-report scales and a subsequent structured interview that elaborates only on the self-report items patients responded to. In this study, all patients received the complete SKID-II interview irrespective of their self-reports. So-called comorbidity, i.e. several personality disorders in one patient, is a known problem of classifications systems [23]. In the present population, a majority of patients were ‘comorbid’ in this sense; the PD category with the highest value in the interview was chosen as the primary diagnosis.

Patients were assigned to the study from three sources, the external psychiatry services Liestal, the psychotherapy day hospital of the University Hospital of Psychiatry Bern, and a number of psychiatrists who worked in private practice in the regions of Bern or Liestal, Switzerland. Assignments were made on the basis of the following inclusion criteria: age ranging from 18 to 55 years, IQ>90, no acute suicidal or aggressive tendencies, no current alcohol or drug abuse, no organic brain dysfunction, no acute axis I symptomatology requiring treatment (such as severe major depression), no predominant post-traumatic stress symptoms. In consistence with the ethics approval of the study, patients with psychotic symptoms, strong dissociative experiencing, suicidality, and self-injuring behavior were excluded from the treatment groups, as symptom-oriented inpatient treatment is considered more appropriate for the stabilization of these presenting issues. Among assigned patients there were no patients with antisocial PD and no patients with acute post-traumatic stress disorder. No further exclusion decisions due to acute symptoms were made once patients were included in the study.

127 patients with a PD diagnosis were assigned to the study groups in total. Two of these patients had received a cluster A diagnosis (this cluster contains the ‘odd’ or ‘eccentric’ disorders: paranoid, schizoid, and schizotypal PD); 81 patients were from cluster B (i.e., the ‘dramatic’, ‘impulsive’ disorders: antisocial, borderline, histrionic, and narcissistic PD); 44 patients were from cluster C (i.e., ‘anxious’, ‘fearful’ disorders: avoidant, dependent, and obsessive-compulsive PD). From this intent-to-treat population of 127 patients, 25 discontinued treatment early, a majority of discontinuations occurred in the control condition (70%; Chi^2^ = 13.0, p<0001). Further 11 patients failed to fill out session reports, leaving 96 patients with full process recordings that were available for the present process study (cluster A diagnosis, 1; cluster B, 60; cluster C, 35). It was decided to exclude the sole patient with cluster A diagnosis for reasons of diagnostic homogeneity, so that 95 patients were finally considered in the present study sample. 69 of these patients were from an efficacy study of schema-centered emotive-behavior therapy (SET) [24], where they were consecutively assigned either to SET or the control condition. 26 patients were from an extension study in which additional groups only of the SET modality were run.

The mean age of patients was 40.5 y; 56 (58.9%) were women. Of all patients, 60 (63%) had received a cluster B diagnosis, 35 a cluster C diagnosis. We tested for biases that may have resulted from patients' discontinuations and the excluded cluster A patient by comparing the study sample (n = 95) with the unstudied sample (n = 32): A likelihood ratio test of the distribution of clusters B and C in these samples was not significant; yet the proportion of female patients was higher in the unstudied sample (81%; Chi^2^ = 5.6, p<05). The Global Assessment of Functioning Scale (GAF = 51.1 in the unstudied sample vs. 52.6 in the studied sample) and the global score of the Brief Symptom Inventory (BSI = 71.2 vs. 77.1) showed no significant difference of functional adaptation or general psychopathology between the two samples.

The largest diagnostic subgroups in the study sample were narcissistic PD (35 patients), borderline PD (20 patients), anxious-avoidant PD (16 patients) and dependent PD (10 patients). Further diagnoses in the sample were obsessive-compulsive PD (6 patients), histrionic PD (5 patients), depressive PD (2 patients) and passive-aggressive PD (1 patient). The distribution of diagnoses in SET and the control therapy condition was not significantly different (Chi^2^ = 5.6; df = 7; p = 0.59).

### Ethics

Participants were informed about the study and gave written informed consent consistent with Swiss ethics regulations. The study was reviewed and approved by the ethics committee of the Canton of Bern.

### Group psychotherapy

Two group psychotherapy treatments were provided to the patients in this study: The specific treatment approach Schema-centered emotive-behavior therapy, SET, was received by 69 patients. The control condition (26 patients) consisted of social skills training (SST) [25].

SET was based on developmental ideas in the assumption that personality disorders are characterized by maladaptive schemas [26]. SET integrates Millon's personality theory [27], concepts of attachment theory [28] and interpersonal theory [29] into schema-theoretical treatment. In this integrative approach, PD are viewed as dysfunctions of interpersonal relations that have emerged from frustrated basic needs. The ensuing relational experiences generate cognitive representations of significant others which become stabilized as negative self-schemas and negative relational schemas.

The therapeutic procedures of SET build on these theoretical assumptions, focusing on activation and clarification of a patient's specific negative core schemas, together with the interactional and intrapersonal strategies associated to core schemas. A short psychoeducative introduction is offered to activate the schemas, followed by hypothetical case reports illustrating each PD. These case reports were specifically designed to reflect the schemas and the linked dysfunctional coping strategies and disorder-specific triggers of crises; they form the essential instruments of the SET approach. Interventions are insight-oriented, focusing not only on the individual strategies but also on their developmental origins. The goal is to illuminate core schemas and strategies as the central components of a vulnerable self, which may then be increasingly experienced as self-dystonic. The therapeutic bond focuses complementarily [30] on establishing and consolidating an autonomous self. In addition to this clarification-based work, cognitive, experiential and behavioral techniques [31] are used.

The effectiveness of the SET approach was previously tested in a controlled evaluation study [13], which showed higher improvements for SET than SST specifically in interpersonal behavior, psychopathology and psychosocial functioning. SET encountered fewer discontinuations than the control treatment. For the purpose of the present process study, we used the comprehensive corpus of session reports of the evaluation study. These data were previously unanalyzed, as well as the complete data from therapy groups that were conducted in the extension to the evaluation study.

The control condition (social skills training, SST) was implemented as a group therapy with problem-centered behavioral elements, yet leaving aside the disorder-specific, schema-oriented approach of SET. SST used standardized exercises described by the established manualised trainings [25], [32]. SST is based on role-playing that covers three types of situations (self-assurance, social relationships, being likeable). Role-plays are repeatedly exercised with supportive feedback given by group members.

Both SET and SST had durations of 30 sessions (100 minutes each) that were conducted twice a week in diagnostically mixed groups of 7–10 patients. All sessions were held in the facilities of the University Hospital of Psychiatry in Bern or the external psychiatric services Liestal, both located in Switzerland. All SET groups were led by a senior psychotherapist and a co-therapist; the former was also the leading author of the SET therapy manual. Allocation to SET or the control treatment SST was not randomized. Allocation was based on the date of referral to therapy: For a given time period, all incoming patients were assigned either to SET or SST, whichever therapy group was open at that time. Possible pharmacological treatment was kept constant throughout the duration of group therapy, and no additional specific psychotherapy was allowed. All groups were conducted by psychologists and/or psychiatrists with training in behavioral psychotherapy. The adherence to the protocol was regularly controlled by an external supervisor.

### Outcome measures

Measures of treatment outcome were calculated by comparing assessments prior to therapy sessions (T1) and directly after termination of psychotherapy (T2). They addressed various domains: psychopathology, interpersonal problems, emotion regulation styles, overall functioning and cognitive patterns. For the purpose of relating process models to outcome at T1 and T2, we used the following instruments: the Constructive Thinking Inventory, CTI-K (German short form with three scales; [33]); the Inventory of Interpersonal Problems, IIP-D (eight scales; [34]); the Global Assessment of Functioning scale, GAF (one scale; [35]); the questionnaire of emotion regulation, EMOREG (four scales; [36]); the Brief Symptom Inventory, BSI (nine scales; [37]); the Questionnaire of Changes in Experience and Behavior, VEV (one scale; [38]). With the exception of the GAF, all instruments were based on self-report. Outcome was defined as the differences of outcome measures between T1 and T2 (with the exception of the VEV, which is a direct change questionnaire applied once at T2). We computed these differences by either subtracting T2 from T1 or vice versa, so that positive values represented improvement on the respective measure. In this way, we defined 26 different outcome scores.

### Measures of therapy process

Therapy process was monitored using therapy session reports of all individual sessions, assessed from the patient's observational perspective. Patients filled out the session reports immediately after each therapy session. The session-report instrument contained 27 items with seven-point Likert scales [39], [40]. We applied principal component analysis, PCA, to condense the 27 session report items into a set of four components. These consequently served as the basis for the time series analyses using Time-series panel analysis [6]. We performed PCA with rotation using Varimax (JMP 9 statistical software for the Macintosh). PCA was applied using all session reports sampled in a larger dataset (2,446 reports of 102 patients, including patients who discontinued treatment). The four-component solution explained 58.9% of total variance; the rotated component scores were obtained by the linear composite of the weighted items, with imputation of single missing data. Through this step of data analysis, the original 27-variate session report data were reduced to four-variate time series of uncorrelated components. These components, with loading scores shown in Table 1, operationalize prominent common factors of psychotherapeutic change [1]. The components (with explained variances) can be described as follows:

**Table 1:** Loadings of components.

session report item:	Clarification	Bond	Rejection	Emotional Activation
Pat1 (I felt well in relationship)	0.17	**0.69**	−0.25	0.30
Pat2 (I understand myself/problems better)	**0.75**	0.13	−0.15	0.33
Pat7 (therapist and I get along well)	0.11	**0.77**	−0.24	0.22
Pat8 (therapist should pay more attention to my feelings)	−0.13	−0.24	**0.72**	−0.06
Pat9 (therapist interested in my well-being)	0.12	**0.77**	−0.15	0.16
Pat11 (I feel better able to solve problems by myself)	**0.84**	0.19	−0.01	0.06
Pat12 (therapist holds a simplistic view of my problems)	−0.07	−0.36	**0.68**	−0.09
Pat13 (I now know better what I want)	**0.82**	0.16	0.02	0.08
Pat15 (I was strongly involved emotionally)	0.23	0.12	−0.04	**0.76**
Pat16 (therapist estimates me)	0.16	**0.79**	−0.09	0.20
Pat17 (I was deeply moved by today's session)	0.23	0.11	−0.01	**0.77**
Pat18 (I feel able to cope with difficult situations)	**0.83**	0.11	−0.02	0.18
patg1 (we really acted as a group today)	0.07	0.40	−0.23	**0.53**
patg4 (group should have been more responsive to my needs)	−0.11	−0.06	**0.78**	−0.03
patg5 (I could not present my issues to the group)	−0.16	0.03	**0.56**	0.08

Data are component loading scores, resulting from principal component analysis (with Varimax rotation) of patients' session reports. Highest loadings are printed bold. Only representative session report items are listed (item content in brackets).

- Clarification (18.5%): Patients experience that they have insight into themselves and their problems. Patients indicate that they know why they act as they do, and that they are clear about individual goals. They feel more self-efficacious, in a position to solve problems.
- Bond (16.3%): High values of this component express the presence of a positive therapeutic relationship and mutual understanding between therapist and patient. The patient feels appreciated by the therapist.
- Rejection (12.3%): A patient loading high on this component refers to a session where he/she feels socially rejected and/or neglected by the therapist as well as the group. The group climate is perceived as aggressive and tense. The patient feels restrained in expressing what moves him/her.
- Emotional Activation (11.7%): A patient feels emotionally aroused, is touched by what has happened in a session and has reached the core of his/her problems. The valence of these emotions may be negative and/or positive. This component includes the patient's assessment of intensified group processes, together with the perception of being integrated as a group member.

These components consequently served as the basis for the time series analyses using Time-series panel analysis, TSPA [6].

### Time-series panel analysis (TSPA)

The multiple time series of components (one four-variate time series for each of the 95 patients) described the complete therapy courses of patients. In cross-section, the components were uncorrelated since they were determined by PCA with orthogonal rotation. Time series analysis, however, exploits the *time-lagged* correlational structure of the components. Accordingly, 95 such time series analyses were independently performed (one in each patient) and then aggregated using TSPA [6]. The mean number of sessions by patient was 25.2 (SD 6.2).

For time series analysis, the procedure VARMAX of SAS® software was applied (SAS Institute Inc., Cary, USA). This method is called vector autoregression (VAR) because each time step of the observed process is given by a vector composed of four scalar variables (here, the scores of Clarification, Bond, Rejection, and Emotional Activation). We used lag-1 VAR models determining the association of each vector (composed of the four components) at any therapy session (i.e., at time t–1) with the vector at the subsequent therapy session t. VAR thus includes regressions of each component to each lagged other component (for instance, the association of Clarification at session [t–1] with Bond at session [t]), as well as four autocorrelations (for instance, Clarification [t–1] → Clarification [t]) that denote the impact each component has on itself at the subsequent time point. Hence, each patient's times series model is represented by 4×4 = 16 parameters. A prerequisite of time-series analysis is that time series are stationary; therefore, linear trends were computed and, prior to VAR, the time series were detrended. In the context of psychotherapy, linear trends are informative as they show which of the components have undergone significant changes of level in the course of therapy.

VAR parameters are estimators of so-called Granger causality [41]. If, for instance, the parameter Clarification [t–1] → Bond [t] has a significant positive value, we may say that Clarification entailed (enhanced, induced) Bond; negative values would suggest that Clarification reduced (inhibited, attenuated) Bond. We use such causal language with the caveat that Granger-causality is merely an approximation of causality.

**Figure 1:**
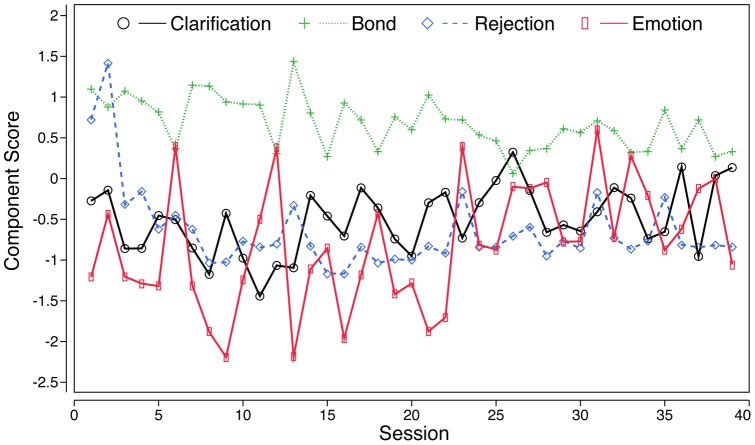
Exemplary time series. Time series of patient #124 (57 y, male, diagnosed as having narcissistic PD). Abscissa, subsequent sessions; Ordinate, values of components Clarification, Bond, Rejection, and Emotional Activation (‘Emotion’).

**Figure 2:**
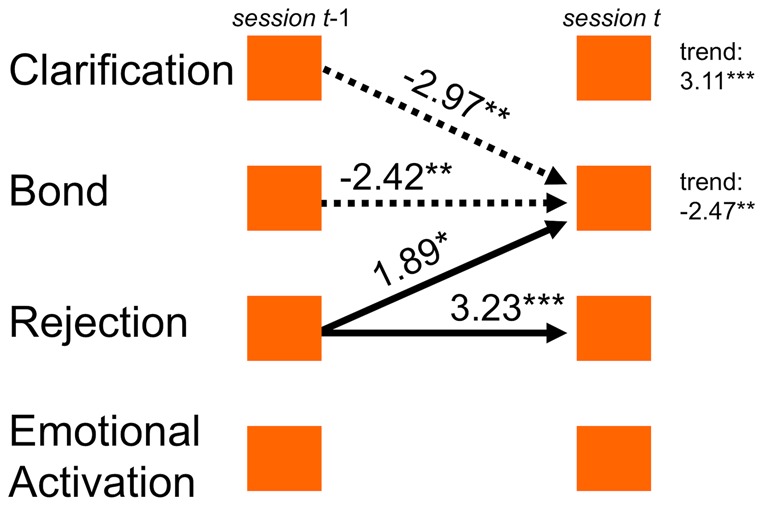
Process model of the time series in Figure 1. Process model of the time series illustrated in Figure 1. Arrows indicate significant time-lagged associations between components identified by vector autoregression analysis. Parameter values are printed next to respective arrows (*, p<.05; **, p<.01; ***, p<.001), significant linear trends are printed right. Negative associations have dotted arrows.

To illustrate the VAR step of the analysis, we show the time series of one patient (Figure 1) and the corresponding time series model of this patient (Figure 2). The model indicates that Rejection at one session enhanced the therapeutic bond at a consecutive session, whereas Clarification reduced this patient's bond with the therapist. Bond was negatively, Rejection was positively autocorrelated. Over the complete course of therapy, this patient showed a significant positive trend of the clarification component, but a negative trend of his therapeutic bond.

The final step of the TSPA procedure – after estimation of all patients' individual process models – was aggregation. Aggregation is the step within TSPA by which the idiographic information of single patients is made accessible to nomothetic testing [6]; this may be performed with respect to the complete sample of patients or specific subsamples. For each of the VAR parameters, a one-sample t statistic was used to test if the (sub)sample's mean parameter values deviated from H_0_ (two-sided testing, p<.05). H_0_ stated that VAR parameters, which can take positive or negative values, would be zero. The models of the individual patients were thus aggregated, yielding sample models such as those of all SET-treated patients or of diagnostic subsamples. A sample model comprises all those parameters that deviate significantly from the expected zero value.

### Process-outcome analysis

Consistent with the focus on process, we explored the association between therapy-induced changes and the process models computed by TSPA. The process-outcome analysis was restricted to patients who had received the specific schema-centered treatment. Outcome measures were available for 66 of all 69 SET patients because three patients had not filled out questionnaires at T2. Outcome was defined as the differences of 26 measures at T1 and T2, where positive values represent improvement on the respective measure. For each outcome score, a regression analysis was performed where the set of 16 times-series parameters acted as predictors and the respective outcome score as the dependent variable. Due to the exploratory nature of this approach and the high number of significance tests involved in it, we did not analyze these regression analyses in any detail. Rather, we adopted in a ‘data-mining’ approach and determined which of the times-series parameters were significant (p<0.05) predictors, and if so, whether they had positive or negative beta weights (i.e., if they contributed to outcome positively or negatively). We then counted the numbers of significances of each parameter to explore the specific impact of process on outcome.

## Results

**Figure 3:**
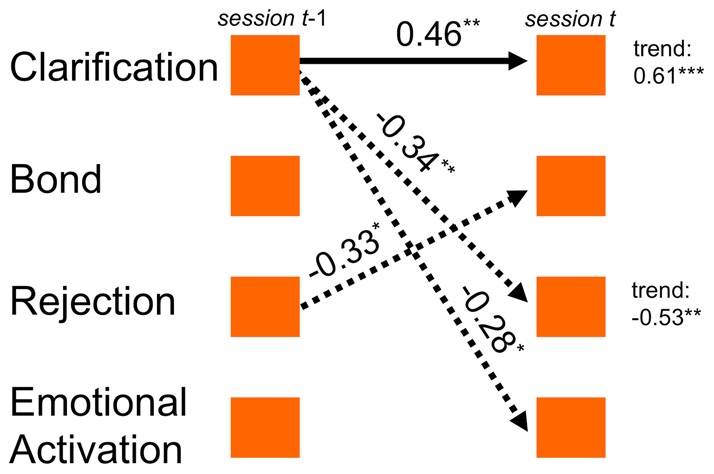
Process model of the SET patients sample. Process model of the SET patients sample (n = 69), after aggregation by TSPA. Arrows indicate those time-lagged associations between components whose sample means deviate significantly from zero. Mean parameter values printed next to respective arrows (*, p<.05; **, p<.01). Negative associations: dotted arrows. Significant linear trends are printed right.

After completion of TSPA, we found two different process models for the two treatment conditions, schema-centered emotive-behavior therapy, SET, and the control treatment, social skills training, SST. These models were derived from the significant parameter values listed in Table 2. The SET process model is illustrated in Figure 3: SET patients were characterized by a dense network of time-lagged associations, with Clarification as the main focus: High clarification values were followed by lowered rejection feelings of patients, and also by their lowered emotional involvement in the group. The clarification component was autocorrelated, i.e. temporally stable. Rejection inhibited the bond between patient and therapist. In the SST condition (not depicted), only one of the lagged relationships was significant: Rejection induced Emotional Activation in the next session. In contrast to these differences of therapy process, the overall linear trends were consistent in both therapy conditions: SET as well as SST showed significant increases of Clarification and decreases of Rejection in the course of therapies. Aggregated trends of Bond and Emotional Activation were not significant. Taken together, these results were consistent with the hypothesis that clarification would play a dominant role in SET.

**Table 2:** Time-lagged parameters.

		SET (n = 69)		SST (n = 26)	
Session t-1	Session t	M	SD	M	SD
Bond	Clarification	0.17	1.17	−0.27	1.44
Rejection	Clarification	0.21	1.00	0.21	1.40
Emotional Activation	Clarification	0.03	1.24	−0.12	1.37
Clarification	Bond	−0.06	1.05	0.03	1.21
Rejection	Bond	−0.33*	1.15	−0.06	1.28
Emotional Activation	Bond	−0.07	1.15	0.34	1.36
Clarification	Rejection	−0.34**	1.05	0.09	1.02
Bond	Rejection	−0.04	1.18	−0.11	1.11
Emotional Activation	Rejection	−0.17	1.21	0.25	0.96
Clarification	Emotional Activation	−0.28*	1.05	0.06	0.84
Bond	Emotional Activation	−0.19	1.02	−0.13	0.77
Rejection	Emotional Activation	0.22	0.94	0.34*	0.83
Clarification	Clarification	0.46**	1.30	0.25	1.43
Bond	Bond	0.18	1.25	0.03	0.81
Rejection	Rejection	0.10	1.33	0.22	1.15
Emotional Activation	Emotional Activation	−0.18	1.06	−0.10	0.74

Data are T-values of associations computed by VAR. Four rows at the bottom of Table 2 denote autocorrelations. M, mean T-value; SD, standard deviation; SET, Schema-centered emotive behavior therapy; SST, social skills training (*, p<.05; **, p<.01). Reading instruction for the first row of Table 2: In the SET group, the bond component at session t-1 is positively, yet not significantly, associated with the clarification component at session t. The mean value of this association in SET patients is 0.17.

**Figure 4:**
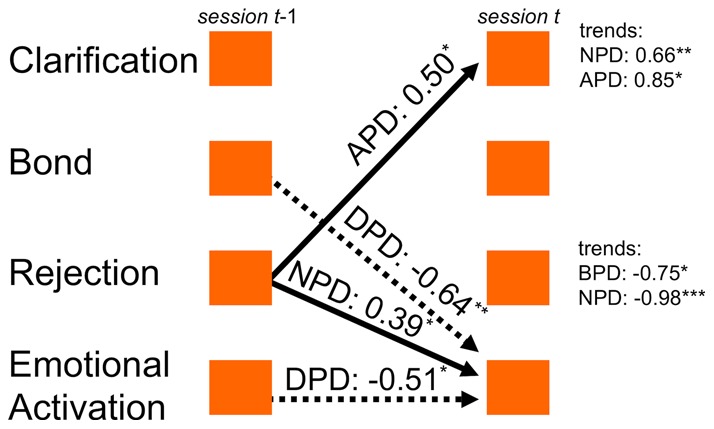
Significant time-lagged associations in diagnostic subsamples. Significant time-lagged associations in diagnostic subsamples: BPD, borderline PD (n = 20); NPD, narcissistic PD (n = 35); APD, anxious-avoidant PD (n = 16); DPD, dependent PD (n = 10). Arrows indicate those time-lagged associations whose means deviate significantly from zero in the respective subsample. Mean parameter values printed next to arrows (*, p<.05, **, p<.01). Negative associations: dotted arrows. Significant linear trends of the single components are printed right.

TSPA was applied to diagnostic groupings of the complete sample (control patients included) by computing process models of diagnostic subsamples. Patients were diagnostically heterogeneous and had received eight different PD diagnoses in structured SCID-II interviews. We analyzed the process models of the four largest subsamples: NPD, narcissistic PD (n = 35); BPD, borderline PD (n = 20); APD, anxious-avoidant PD (n = 16); DPD, dependent PD (n = 10). All time-lagged associations with significant mean values are depicted in Figure 4. In addition to PD-specific time-lagged (i.e. session-to-session) associations of the four process components, we also considered trends, i.e. linear increases or decreases of the components in a diagnostic subsample, which occurred over the entire course of therapy. We found APD characterized by a positive parameter Rejection [t–1] → Clarification [t] and by a significant linear trend (i.e. increase) of the clarification component. The (small) DPD subsample showed negative autocorrelation of Emotional Activation and a reduction of Emotional Activation by the therapeutic bond component. NPD patients presented a specific pattern in that Rejection enhanced their Emotional Activation. NPD patients had significant trends of two components over the period of treatment: increase of Clarification and decrease of Rejection. BPD had no significant lagged parameters but showed also a decrease of Rejection.

### Process-outcome analysis

In the sample of SET patients, we determined time-lagged parameters that were linked with outcome. The exploratory nature of the process-outcome analysis consisted of estimating the predictive value of 16 time series parameters in each of the 26 outcome measures. Thus, 16×26 = 416 beta weights were computed in 26 regression analyses. Of the 26 regressions, four resulted in significant (p<.05) process-outcome relations and four more indicated possible process-outcome relations at the trend level (p<.1). In the four significant regression models, 14 predictors with significant beta weights (‘hits’) were found (Table 3).

**Table 3:** Process-outcome analysis.

Dependent variable:		CTI global score (n = 66)	CTI emotional coping (n = 66)	IIP introverted (n = 66)	IIP overprotective (n = 1413)
significant predictors	Clarification → Clarification				
	Bond → Clarification				
	Rejection → Clarification				
	Emotion → Clarification				
	Clarification → Bond				
	Bond → Bond				
	Rejection → Bond				
	Emotion → Bond		−2.49*	−3.16**	−2.53*
	Clarification → Rejection				−2.39*
	Bond → Rejection				
	Rejection → Rejection				
	Emotion → Rejection		2.42*	2.48*	3.06**
	Clarification → Emotion	−2.02*	−2.66*		
	Bond → Emotion			2.13*	
	Rejection → Emotion				
	Emotion → Emotion	−3.49***	−3.10**	−3.18**	−2.95**
				
Whole model	Explained Variance (% of Total)	38.1	39.3	38.5	44.3
	F ratio	1.88*	1.98*	1.92*	2.44**

Multiple regression models of outcome scores (dependent variables) predicted by time series parameters. Only significant regression models are shown.

* *p<*.05; ** *p<*.01; *** *p<*.001.

CTI, Constructive Thinking Inventory; IIP, Inventory of Interpersonal Problems; Emotion, Emotional Activation.

**Figure 5:**
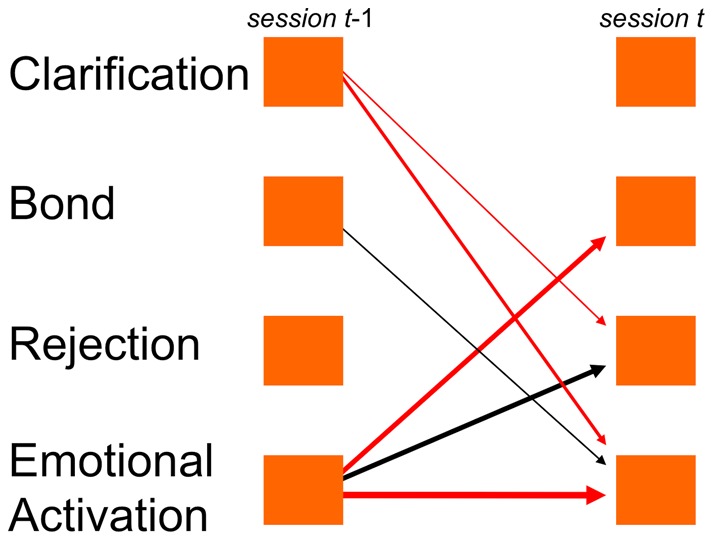
Time-lagged associations with significant links to outcome. Time-lagged associations with significant links to outcome (SET patients). Black (red) arrows indicate those associations that predicted positive (negative) outcome. Thickness of arrows indicates the number of ‘hits’ (cf. Table 3).

These predictors originated from a group of six time series parameters as several parameters were repeatedly found among hits; they are depicted in Figure 5. All outcome-related parameters were connected with Emotion or Clarification, at either the donating or receiving end of time-lagged associations. Negative autocorrelation of the Emotional Activation component predicted favorable outcome (four hits). The same was true when Emotional Activation reduced Bond (three hits) and enhanced Rejection (three hits). Both time-lagged associations of Clarification already found to characterize the SET process (Figure 3) were outcome-related: if Clarification inhibited Rejection (two hits) and Emotional Activation (one hit), outcome was improved. The parameter Bond [t–1] → Emotional Activation [t] was positively linked with outcome (one hit).

## Discussion

The motivation of the present study was to explore change mechanisms of psychotherapy of personality disorders (PD). We therefore focused on temporal, time-lagged patterns when analyzing a dataset that depicts the process of group psychotherapy of outpatients with PD. In the dataset, process data of an efficacy study of schema-centered emotive-behavior therapy, SET, were merged with a consecutive extension study using SET in ambulatory patients. The study provided a detailed quantitative representation of therapy process on the basis of a novel time-series approach, time series panel analysis (TSPA). TSPA elaborated the mechanism of action of the schema-centered therapy approach, and showed process-related differences between patients from different PD categories. TSPA also indicated which specific process components were linked with therapy outcome.

All computations of temporal patterns rested on components (Clarification, Bond, Rejection, and Emotional Activation) that were derived from patients' session reports; these components reflect four essential aspects of each therapy session. Clarification, Bond and Emotional Activation are closely related to established common factors of psychotherapy research [42], [17]. Rejection and Emotional Activation cover affective states that are of obvious importance to PD patients in therapy groups, where patients are prone to experience interpersonal problems and emotional dysregulation. Hence, the process model of SET (Figure 3) shows a temporal mechanism of action that is consistent with the philosophy of schema-centered therapy: Clarification (with this component's connotations of insight into maladaptive schemas) served to inhibit the rejection PD patients experienced in the therapeutic situation. Rejection reduced Bond, i.e. perceived social rejection reduced the patients' perceived alliance with the therapist. Clarification also inhibited and limited emotionality. It was found that Clarification was autocorrelated, i.e. temporally stable throughout sessions. This sketch of therapeutic mechanisms in this study appeared plausible and well in accordance with the change mechanisms not only proposed by schema therapy but also by insight-oriented approaches in general. The results furthermore indicate that TSPA methodology can have considerable worth for explicating mechanisms of action in psychotherapy research.

The application of TSPA to PD subgroups highlighted those time-lagged associations that were specific to diagnoses (Figure 4). This analysis was limited owing to the small sample sizes, especially in anxious-avoidant (APD) and dependent PD (DPD). APD patients were characterized by Rejection enhancing Clarification, which may be interpreted along the lines of exposition to socially aversive stimuli supporting insight in these patients. Narcissistic PD patients reacted differently to Rejection, namely by enhanced emotionality. The key role of social rejection in both anxious and narcissistic personality styles is well in line with the phenomenology of these styles: both anxious and narcissistic persons are highly sensitive to situations of perceived interpersonal rejection, to which they then respond differently. Emotional Activation was relevant also for patients with DPD: they showed fluctuating, unstable emotionality (negative autocorrelation of Emotional Activation) together with Bond inhibiting Emotional Activation. Although we have to acknowledge the preliminary nature of the present findings, these temporal characteristics appear consistent with clinical descriptions of anxious-avoidant, narcissistic, and dependent personality styles.

The third area of investigation addressed the therapeutic process-outcome relationships. The parameters depicted in Figure 5 suggested that the components Emotional Activation and Clarification played major roles with respect to outcome in SET. These results indicate that specific parameters – which described the inhibitory action of Clarification with respect to Rejection and Emotional Activation in the SET process model (Figure 3) – were also predictive of favorable outcome. Thus, the emphasis put on insight and clarification in the development of schema-oriented psychotherapy approaches appears empirically supported by the analysis of outcome. A further aspect of process-outcome relationships is worth mentioning: There is an interesting reciprocal exchange between Bond and Emotional Activation, which is reminiscent of a negative feedback loop. ‘Bond enhancing Emotional Activation’ together with ‘Emotional Activation inhibiting Bond’ is linked to positive outcome. This reciprocal connection, if present in a patient or therapy group, may have self-limiting function (as is the case in negative feedback systems in cybernetics). This function may help Bond and Emotional Activation to remain within their bounds of effectiveness.

Emotional Activation was largely involved in those parameters that were predictive of outcome (Figure 5). This involvement appears well in line with the significance of emotionality in the treatment of PD, yet counterintuitive in two instances: Why were both emotional enhancement of Rejection and emotional inhibition of Bond predictors of good outcome? One possible explanation is that Emotional Activation may have been instrumental for the staging and actualization of a patient's conflicts and problems in the therapeutic context, and that this process was beneficial for outcome. This is reminiscent of the dialectics between problem instigation and resource activation claimed by psychotherapy theorists [8], [17].

### Limitations

A limitation of this study is its partly descriptive and, with respect to process-outcome analysis, exploratory nature. The exploratory nature is apparent in the fact that no correction for multiple testing was introduced. It would be preferable to formulate process hypotheses throughout, e.g. on the schemas underlying specific disorders (e.g., [10]), and consequently test these hypotheses using the process data. The validity of the process data we used depended on patients' self-reports: Thus, when speaking of, e.g., clarification, we always refer to what a patient considers more or less clarified, but there is no way of knowing objectively whether real clarification and insight into a schema has occurred or not. This problem may be specifically enhanced in PD patients whose self-reports tend to be biased. Other limitations rest in the diagnostic problems inherent to DSM classification. Diagnoses of PDs can be inconsistent across clinicians, and individuals can have more than one diagnosis, the so-called comorbidity [43] of psychiatric classification systems. It should be noted that the PD patients of this study sample do not represent all PD patients in psychiatric treatment. Due to its outpatient setting, this study only included patients whose symptoms could be managed in an ambulatory or day-hospital context. As process variables, we used PCA of all session reports available in the present project. Thus, the resulting components are valid only in the context of this dataset, and components may be constructed differently in other datasets and with other diagnoses. The present ad hoc PCA, however, generated components that appeared congruent with common factors known from psychotherapy process research.

An important caveat connected to time-series analysis is that Granger causality is an approximation of causality on the basis of temporal sequence. Yet in principle other, unobserved variables may have contributed to the significant effects. It is therefore mandatory to keep in mind that the causal wording we have used in this article is to be understood in a Granger-causal context. It must also be considered that the time-lagged associations rely on session-to-session time steps. It is not known which associations may occur at other time lags (e.g. within a session) simply because such fine-grained measurement was not available. Finally, we have to point out the limited generality of process models in subsamples because of the differing, and in some diagnostic groups insufficient, sample sizes. Lack of testing power thus ruled out modeling of subsamples in the SET and control condition separately.

In conclusion, we based our analysis on the fact that the essence of psychotherapy is process, and that temporal patterns are likewise implied in the definitions of personality disorders. Psychotherapy process contains the active ingredients of psychotherapy, its mechanisms of action and the factors that produce change. On this background, we propose to apply time-series analysis because it conceives of process and pattern as significant time-lagged association. This methodology was shown to generate plausible models of how a specific schema-centered therapy approach becomes functional, and what its outcome-relevant components are. It can also generate suggestions for specific process patterns underlying specific diagnoses of PD.

We consider this temporal conception of pattern as being superior to cross-sectional concepts, which can only indirectly represent core concepts of psychotherapy (such as ‘change mechanism’) and of personality psychopathology (such as ‘enduring pattern’). If theoretical concepts are temporal, so should be the instruments of inquiry. Our study has demonstrated that this methodology is feasible when appropriate process data are available. In comparison with cross-sectional RCTs, we think that the value of temporal methods lies not only in substituting RCTs when these cannot be applied for practical reasons. Rather, temporal methods constitute an alternative model of investigation of causal mechanisms that is better suitable for shedding light on core aspects of such mechanisms.

We therefore invite researchers in the field to make use of session reports and related measures that can describe therapy process in a fine-grained fashion. Process research can be put to many causes: it allows to check for therapy fidelity; it can highlight the modes of action by which treatment becomes effective; it allows to investigate the actual patterns that characterize specific diagnoses, guiding intervention to better adapt to these patterns.
